# Calcium channel blocker overdose: Experience with amlodipine

**DOI:** 10.4103/0972-5229.45080

**Published:** 2008

**Authors:** Supradip Ghosh, Mrinal Sircar

**Affiliations:** **From:** Fortis-Escorts Hospital, Neelam Bata Road, Faridabad, Haryana-121 001, India

**Keywords:** Amlodipine overdose, calcium, calcium channel blocker, glucagon, hyperinsulinemic euglycemia

## Abstract

Amlodipine overdose is only scarcely reported from India. We report two cases of near fatal Amlodipine overdose managed in our ICU with fluid, vasopressors, calcium infusion and Glucagon. Literature is reviewed and other treatment modalities discussed.

## Introduction

Calcium channel blockers are the leading cause of cardiovascular drug overdose and are responsible for 48% of deaths related to cardiovascular drug exposure.[1] Treating patients with overdose of these medications can challenge even the most experienced physician. The difficulty arises because patients severely poisoned with calcium channel blockers may have profound refractory bradycardia and hypotension.[1] Reports of calcium channel blocker overdose are scarce in Indian literature. We report two cases of Amlodipine overdose treated in our ICU.

## Case Reports

### Case 1

A 25-year-old physiotherapist was brought to our hospital with complaints of recurrent vomiting following ingestion of 100 mg of Amlodipine and few sustained release tablets of Diclofenac, four hours earlier. Initially she was taken to a local hospital, where gastric lavage was performed and she was treated with intravenous fluids and Dopamine infusion in view of low blood pressure. There was no history of syncope/seizure, dyspnoea or palpitations. She was a known case of bronchial asthma on inhaled Salbutamol as and when required. On examination, in the ICU of our hospital, she was drowsy but arousable on light stimuli. She was pale with cold, clammy extremities, her heart rate was 80/min, regular, sinus rhythm and her blood pressure was 60 mm Hg systolic. There were no heart murmurs or gallop, and her lungs were clear on auscultation. Abdominal examination revealed mild epigastric tenderness. Her initial hemogram, liver & renal function tests, ABG and electrolytes were unremarkable. The serum lactate level was 2.3 mmol/L (Normal reference range – 0.5-2.2 mmol/L). No abnormality was detected in the chest X-ray and electrocardiogram. Echocardiography revealed normal chamber size with normal LV systolic function.

In addition to the standard resuscitative measures, the patient was treated with 30 ml of 10% Calcium Gluconate over 5 min followed by an infusion of 10 ml/hr of calcium gluconate. 10 mg of Glucagon was administered intravenously as a stat dose, and an infusion of Glucagon at 3 mg/hr was continued. Infusions of normal saline (totaling 5.5 L during first 24 hours) and noradrenaline was used to support the blood pressure. With these measures, the patient started showing improvement in her hemodynamics, only to deteriorate after 24h with shortness of breath and clinical/radiological features suggestive of pulmonary edema. Repeat echocardiogram was performed which did not reveal any abnormal findings. The patient was treated with diuretics and oxygen. Over the next 48h she showed gradual improvement in her clinical condition. Inotropes, Calcium and Glucagon infusions were ceased after 72h of admission. On day five, she was discharged in good health after a psychiatry consultation.

### Case 2

A 65-year-old male was admitted to our ICU with a history of restlessness following accidental ingestion of 50 mg of Amlodipine along with his usual dose of 50 mg Atenolol, six hours earlier. He was a known case of hypertension for 15 years, on regular medications. He was diagnosed to have mild renal insufficiency 6 years prior to present admission, with a stable serum creatinine level. On examination he was conscious, oriented with normal sinus rate of 62/ min, blood pressure of 112/76 mmHg and bilateral pedal edema. Respiratory, cardiovascular and neurological examinations were normal. Electrocardiograph showed normal sinus rhythm. Initial hemogram, random blood sugar, serum electrolytes, arterial blood gas and electrocardiogram were unremarkable. Blood urea and serum creatinine values were 79 mg/dl and 4.3 mg/dl respectively. Echocardiography revealed left ventricular hypertrophy with normal LV systolic and diastolic function.

The patient was given 30 ml of 10 % calcium gluconate - over 5 mins, followed by an infusion of calcium gluconate at a rate of 10 ml/hr and after a bolus dose of Glucagon of 10 mgm, an infusion of Glucagon at a rate of 3 mg/hr was commenced. Over the next six hours the patient became hypotensive not responding to volume resuscitation and requiring inotropic support with adrenaline and dopamine infusion. His sensorium gradually deteriorated. Twelve hours following the overdose he was unresponsive to painful stimuli. Arterial blood gas analysis revealed mixed respiratory and metabolic acidosis with a pH of 6.8, pCO_2_ of 115 mmHg, pO_2_ of 76 mmHg and a HCO3 of 16 mmol/L. He was on high dose inotropic support with normal central venous pressure and there was a drop in the hourly urine output. Gastric aspirate was coffee ground. He was electively intubated and ventilated. Ultrasonography of the abdomen showed normal kidney size with increased echogenicity. UGI endoscopy revealed erythematous gastric mucosa without any ulcer crater.

The next day the patient started showing signs of improvement. His sensorium improved but he remained oliguric. Arterial blood gas analysis showed pH of 7.2 pCO_2_ of 34. mm Hg, pO_2_ of 115 mmHg and a bicarbonate of 13.7 mmol/L. Repeat potassium was 7.8 mEq/L. In view of oliguria, persistent acidosis and hyperkalemia hemodialysis was started. Over the next 24h, his condition stabilised and inotropic support, glucagon, calcium infusions were tapered off. He was successfully weaned off from the ventilator on the following day. On day 10 of admission he was discharged from the hospital.

## Discussion

Amlodipine is a dihydropyridine group of calcium channel blockers (CCBs) having a half life of 30-50 hours and a large volume of distribution (21 L/Kg).[1] Unlike nondihydropyridine CCBs like Verapamil and Diltiazem, dihydropyridines as a group have predominant effect on vascular smooth muscle cells with little effect on cardiac pacemaker cells or contractility.[2] But in significant overdose some of this pharmacological selectivity may be lost.[1]

In both the cases described, the effect of Amlodipine on vascular smooth muscle was evident. Both the patients developed profound hypotension requiring prolonged inotropic support without significant effect on cardiac pacemaker or conduction system and preserved systolic function of the heart. The first case was complicated by transient pulmonary edema which might have resulted from the combined effects of the drug itself, prolonged hypotension and fluid resuscitation during the initial phase of therapy. Normal cardiac function on echocardiography, excluded myocardial depression as an etiologic factor. Noncardiogenic pulmonary edema following CCB overdose is well described in the literature.[3–5] Pre-capillary vasodilatation resulting in excessive pulmonary capillary transudation was suggested as the possible mechanism of non-cardiogenic pulmonary edema by Humbert *et al*.[3]

The second patient was complicated by acute on chronic renal failure and had mixed respiratory and metabolic acidosis, requiring mechanical ventilation and hemodialysis. CCB overdose is frequently complicated by renal failure, related to the severe hypoperfusion and end-organ ischemia.[1] Metabolic acidosis in our patient could be attributed to the renal failure and prolonged hypotension, well described in the literature. Decreased insulin secretion and increased insulin resistance may also lead to the metabolic acidosis in CCB poisoning.[5] Additionally, CCB mediated inhibition of calcium-stimulated mitochondrial activity may lead to interference with glucose catabolism resulting in increased lactate production and ATP hydrolysis contributing to the acidosis.[1] Respiratory acidosis complicating CCB overdose has not been described in the past. Only possible explanation for this ventilation failure could be the decreased respiratory drive caused by the cerebral hypoperfusion in the absence of any primary pulmonary pathology.

There is no definitive evidence that gastrointestinal decontamination either in the form of activated charcoal or the whole bowel irrigation alters the clinical outcome in the CCB overdose. However, GI decontamination is still advocated because of the potential lethal nature of this overdose and lack of specific efficacious antidote. But potential risks of GI decontamination should be kept in mind e.g. gastric lavage should probably be withheld in patients who are already bradycardic or have conduction disturbances.[6] There is potential for delayed toxicity by sustained release preparations of calcium channel blockers due to delayed absorption. Case reports have suggested the importance of whole bowel irrigation in these cases.[7,8]

Hyperinsulinemic euglycemia has emerged as possible adjuvant therapy for CCB toxicity. Several possible roles of Insulin are described. Insulin increases plasma levels of ionized calcium, improves hyperglycemic acidotic state, improves myocardial utilization of carbohydrates and exerts its own independent inotropic effect.[9] Currently all available information on hyperinsulinemic euglycemia therapy is limited to case reports and series. Probably it should be considered for patients CCB overdose who do not respond to initial supportive therapy.[9]

Many other treatment modalities have been described in the literature. Transvenous pacing may be required in patients with severe symptomatic bradycardia not responding to Atropine or Isoprenaline infusion.[10] Standard cardiopulmonary bypass has been used in some cases to allow sufficient time for liver detoxification.[11] Extracorporeal membrane oxygenation was described in massive Diltiazem overdose for temporary hemodynamic support.[12] Therapeutic plasma exchange was also utilized in the management of certain cases of Amlodipine overdose.[13] Hemofiltration and dialysis may not be of help in Calcium Channel Blocker overdose because of high protein binding, extensive tissue distribution and rapid rate of metabolism of this group of drugs.[10]

We conclude that Amlodipine overdose can be treated successfully with early GI decontamination, resuscitation with calcium and glucagon infusion, judicious use of inotropes and careful monitoring of possible complications. Prospective trial on the use of hyperinsulinemic euglycemia therapy is required to define its role as the first line treatment in CCB overdose.
